# Structure-based virtual screening identified novel FOXM1 inhibitors as the lead compounds for ovarian cancer

**DOI:** 10.3389/fchem.2022.1058256

**Published:** 2022-11-24

**Authors:** Zi-Ying Zhou, Xiao-Yang Han, Lian-Qi Sun, Si-Yan Li, Si-Tu Xue, Zhuo-Rong Li

**Affiliations:** Institute of Medicinal Biotechnology, Chinese Academy of Medical Sciences and Peking Union Medical College, Beijing, China

**Keywords:** ovarian cancer, FOXM1, virtual screening, camptothecin, inhibitors

## Abstract

Ovarian cancer (OC) is a gynecological tumor with possibly the worst prognosis, its 5-year survival rate being only 47.4%. The first line of therapy prescribed is chemotherapy consisting of platinum and paclitaxel. The primary reason for treatment failure is drug resistance. FOXM1 protein has been found to be closely associated with drug resistance, and inhibition of FOXM1 expression sensitizes cisplatin-resistant ovarian cancer cells. Combining existing first-line chemotherapy drugs with FOXM1 prolongs the overall survival of patients, therefore, FOXM1 is considered a potential therapeutic target in ovarian cancer. Previous research conducted by our team revealed a highly credible conformation of FOXM1 which enables binding by small molecules. Based on this conformation, the current study conducted virtual screening to determine a new structural skeleton for FOXM1 inhibitors which would enhance their medicinal properties. **DZY-4** showed the highest affinity towards FOXM1, and its inhibitory effect on proliferation and migration of ovarian cancer at the cellular level was better than or equal to that of cisplatin, while its efficacy was equivalent to that of cisplatin in a nude mouse model. In this study, the anti-tumor effect of **DZY-4** is reported for the first time. **DZY-4** shows potential as a drug that can be used for ovarian cancer treatment, as well as a drug lead for future research.

## Introduction

Ovarian cancer is a serious health issue that endangers the health of women worldwide. The rate of incidence of ovarian cancer is ranked third among those of gynecological cancers, second to those of cervical cancer and uterine cancer (Momenimovahed, et al., 2019). Globally, 308,069 new cases of ovarian cancer were diagnosed in 2020, resulting in 193,811 cancer-specific deaths (Yang et al., 2020). Ovarian cancer has the highest mortality rate and the worst prognoses among gynecological cancers, with the 5-year survival rate being only 47.4% (Stewart et al., 2019). Due to the absence of formal screening methods and obscure early symptoms, a majority (approximately 80%) of patients are diagnosed at an advanced stage (III/IV) and do not qualify for radical surgery (Gralewska et al., 2020). Although first-line treatment, consisting of surgery followed by combination chemotherapy, usually carboplatin and paclitaxel, is effective (Gadducci et al., 2019), approximately 75% of patients with advanced ovarian cancer experience recurrence within 3 years (Dasari and Tchounwou, 2014). In recent years, despite new advances in drug therapy, such as the combining of bevacizumab, a vascular endothelial growth factor receptor (VEGFR) inhibitor, with carboplatin and paclitaxel chemotherapy, progression free survival (PFS) was prolonged only by 3.5 months, with no significant differences in overall survival (OS) being observed (Lheureux et al., 2019). Recently, poly (ADP-ribose) polymerase (PARP) inhibitors, such as niraparib, became the first targeted agents to be approved by the United States Food and Drug Administration Agency (FDA). PARPs imparted substantial clinical benefits to recurrent ovarian cancer patients following platinum-based chemotherapy, the median PFS in the niraparib group being significantly longer than that in the placebo group (21.9 months vs. 10.4 months, respectively) (Lord and Ashworth, 2017). However, its efficacy is somewhat limited due to the presence of diverse resistance mechanisms, including the loss of PARP1 protein itself, which maintains replication fork stability and mutations in *BRCA* thereby sufficiently restoring the homologous recombination repair (HRR) function (Jiang et al., 2020). Currently, drug therapy remains the mainstay of ovarian cancer treatment, and the need to identify new targets and more effective drugs against FOXM1 in order to improve treatment has been recognized.

Forkhead box M1 (FOXM1), a member of the Forkhead box (FOX) transcription factor family (Su et al., 2021), is well-known for its imperative role in various physiological functions, including cell cycle regulation, DNA damage repair, and apoptosis. It is ubiquitously expressed in proliferating and regenerating mammalian cells. FOXM1 contributes to oncogenesis (Quan et al., 2013), overexpression of FOXM1 increased invasion (Halasi and Gartel, 2013), angiogenesis (Karadedou et al., 2012), epithelial-mesenchymal transition (Meng et al., 2015), and self-renewal as well as chemotherapy resistance (Gomes et al., 2013). The Cancer Genome Atlas (TCGA) revealed that the FOXM1 transcriptional pathway was eccentrically activated in over 85% of high-grade serous carcinoma (HGSC) cases (Pratheeshkumar et al., 2018) and that high FOXM1 expression was associated with the stage and prognosis of ovarian cancer (Gentles et al., 2015). Other studies have shown that FOXM1 enhances the invasion and metastasis of ovarian cancer cells by upgrading the expression of matrix metalloproteinase (MMP-2) and degrading the extracellular matrix (Gentles et al., 2015). In addition, FOXM1 promotes DNA repair and chemotherapy resistance. Thiostrepton, a small molecule inhibitor (SMI) of FOXM1, reportedly sensitizes ovarian cancer cells to platinum-based chemotherapy (Lin et al., 2013). Furthermore, cisplatin-resistant ovarian cancer tissues and cells display increased FOXM1, which therefore acts as an independent indicator of the rapid progression of platinum-resistant epithelial ovarian cancer (EOC); (Tassi et al., 2017). Investigations of FOXM1 also showed that it plays a role in PARPi and paclitaxel resistance in ovarian cancer (Liu et al., 2021). In conclusion, FOXM1 plays an important role in the development of ovarian cancer as well as in drug resistance in this disease, indicating that inhibition of FOXM1 activity may play a role in ovarian cancer therapy.

**FIGURE 1:**
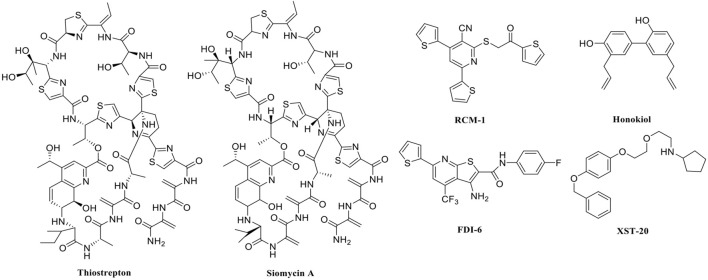
The chemical structure of FOXM1 inhibitors.

In recent years, FOXM1 inhibitors have been successively reported, and the structures of some are shown (Figure 1). Siomycin A and thiostrepton were reported as inhibitors of the transcriptional activity of FOXM1. However, these two are protease inhibitors, which target multiple signaling pathways in addition to those associated with FOXM1 (Bhat et al., 2009). XST-20 was identified as a leading compound that inhibits the transcriptional activity of FOXM1 and specifically blocks FOXM1-DNA binding. However, it poses certain medicine-related issues, such as poor water solubility and oral absorption difficulties (Zhang et al., 2022). Gormally et al. used a high-throughput screen, to demonstrate that FOXM1 is inhibited by a novel small molecule inhibitor, FDI-6, and they also have demonstrated that the ligand makes direct contact with FOXM1 and inhibits DNA binding in cells. However, its high cytotoxicity limited its use in many biomolecular experiments (Gormally et al., 2014). Shukla et al., also identified a novel small molecule, RCM-1, *via* high-throughput screening, which can effectively and specifically inhibit nuclear localization of the FOXM1 protein, causing it to be ubiquitinated and degraded by proteasomes. However, the binding mode of RCM-1, FDI-6, and FOXM1 remains unclear (Shukla et al., 2019). The natural product, honokiol reportedly hinders FOXM1 transcriptional activity, disrupting the FOXM1 auto-regulation loop and reducing FOXM1 mRNA and protein expression. However, further clinical application of these inhibitors is hampered by their insolubility in water and resistance to absorption (Halasi et al., 2018). Currently, there is no explicit FOXM1-based drug lead that can be used in subsequent studies. Our group previously used molecular docking to study the binding mode of FDI-6 to FOXM1. We conducted FDI-6 similarity retrieval to establish a derivative library, following which affinities of the compounds in the derivative library were determined *via* surface plasmon resonance (SPR), at the molecular level, and *via* IC_50,_ at the cellular level. Based on the relationship between virtual docking energy, affinity, and anti-ovarian cancer activity of compounds, a more reliable binding conformation of FDI-6 and FOXM1 was identified.

In this study, we aimed to explore new structural types of FOXM1 inhibitors utilizing previous binding conformations to identify potential drug candidates for ovarian cancer. Our findings may also be used as a biochemical research tool to identify the molecular mechanism underlying the role of FOXM1 in ovarian cancer, as well as to assess its value as a therapeutic target in this disease.

## Results and discussion

### Structure-based virtual screening for FOXM1 inhibitors

**FIGURE 2:**
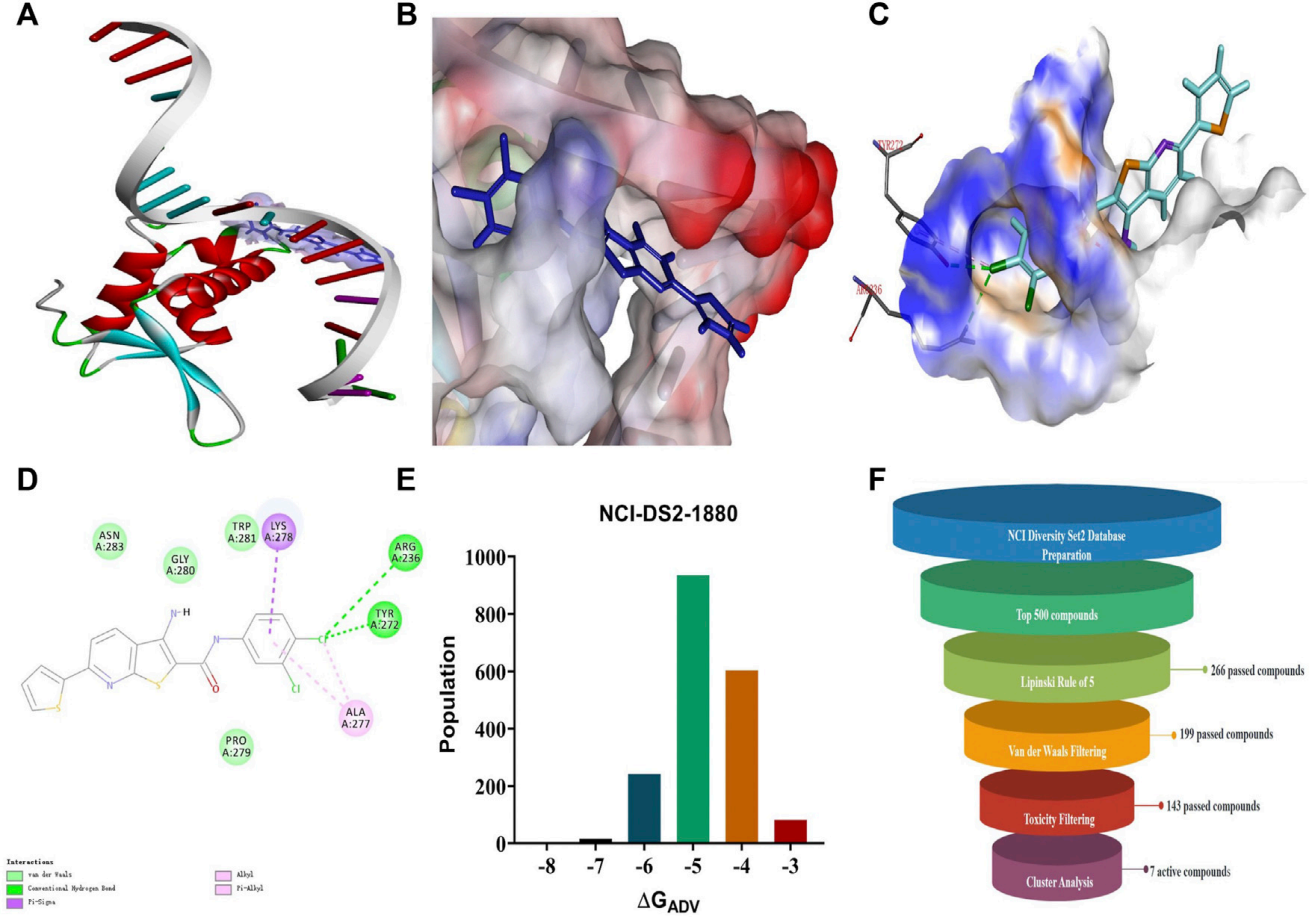
Structure-based virtual screening for FOXM1 inhibitors. **(A)** The position of FOXM1 binding to small molecule. **(B)** Surface view of a small molecule bound to FOXM1 **(C)** Close-up view of a small molecule bound to FOXM1.The key residues that contact the inhibitor are labeled. The small molecule is shown in purple. **(D)** 2D interaction diagram of FOXM1 and the small molecule. **(E)** Results of the VS. (using ADV) of the NCI Diversity Set 2 against FOXM1. Bars represent numbers of Diversity Set compounds with predicted free energies of binding in indicated 1 kcal/mol bins. **(F)** Overview of the flowchart for the discovery of FOXM1 inhibitors.

A search for new FOXM1 inhibitors *via* structure-based virtual screening requires the identification of the cavity in which FOXM1 binds small molecules. The crystal structure of FOXM1 (PDB ID: 3G73) obtained from the RCSB protein database is used to generate receptor input during the docking process. The position at which FOXM1 binds to small molecules, as indicated by previous studies, is shown (Figure 2A). The surface binding mode is also shown (Figure 2B). The key amino acids involved in the binding between FOXM1 and small molecules are suggested to be ARG236, TYR272 (Figures 2C,D; docking pose). We used this binding site for subsequent screening.

First, we used a structure-based virtual docking approach to screen the National Cancer Institute (NCI) Diversity Set 2, which represent the diversity of 265242 compounds available at NCI. A total of 1880 compounds were docked at FOXM1 according to AutoDock Vina (ADV). The virtual screening (VS) results were sorted based on their predicted binding free energies (GADV). The results indicated that ∆GADV ranged from −2.8 to −7. 8 kcal/mol, where 13.7% of Set 2 had energies lower than −6.0 kcal/mol (GADV of FDI-6); (Figure 2E). To narrow down the selection and eliminate unsuitable molecules in the database, we used the calculated binding energies data to select 500 compounds. These compounds were filtered according to Lipinski’s “Rule of five” and the Veber rule. The remaining compounds were further refined using the “toxicity prediction” tool, implemented by the Discovery Studio (DS), to filter out potentially carcinogenic, mutagenic, or teratogenic compounds. This three-step filtering procedure, generated a library containing a 25 specs drug-like set (Figure 2F). Finally, we comprehensively evaluated binding energy data, interactions with key residues at binding sites, and structural diversity to select 7 compounds for subsequent *in vitro* biological evaluation, the DS virtual screening scores of which are reported (Table 1).

**TABLE 1:** Energy values and amino acid interactions of compounds combined with FOXM1.

Name	Structure	Compound	CDOCKER energy (kcal/mol)	H-bond
**DZY-1**	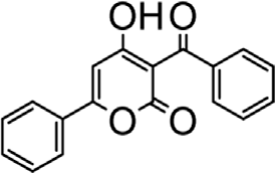	ZINC02476372	−6.2727	ARG236, TYR272
**DZY-2**	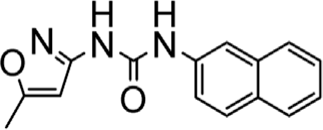	ZINC00031410	−6.2727	ARG236, TYR272
**DZY-3**	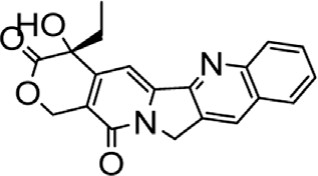	ZINC00001087	30.7268	ARG236
**DZY-4**	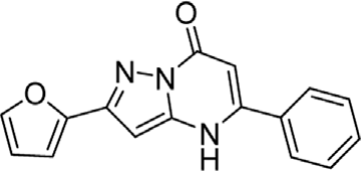	ZINC13597767	−18.1145	ARG236, TYR272
**DZY-5**	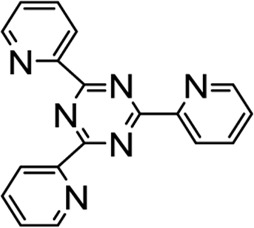	ZINC00039221	−14.0238	ARG236, TYR272
**DZY-6**	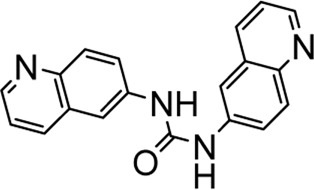	ZINC00393674	−1.9784	ARG236
**DZY-7**	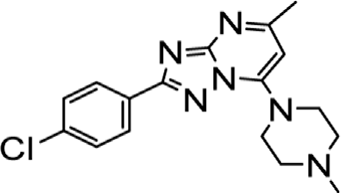	ZINC01556940	−11.939	ARG236, TYR272

### Structure-based virtual screening aimed at discovering and validating FOXM1 inhibitors

**FIGURE 3:**
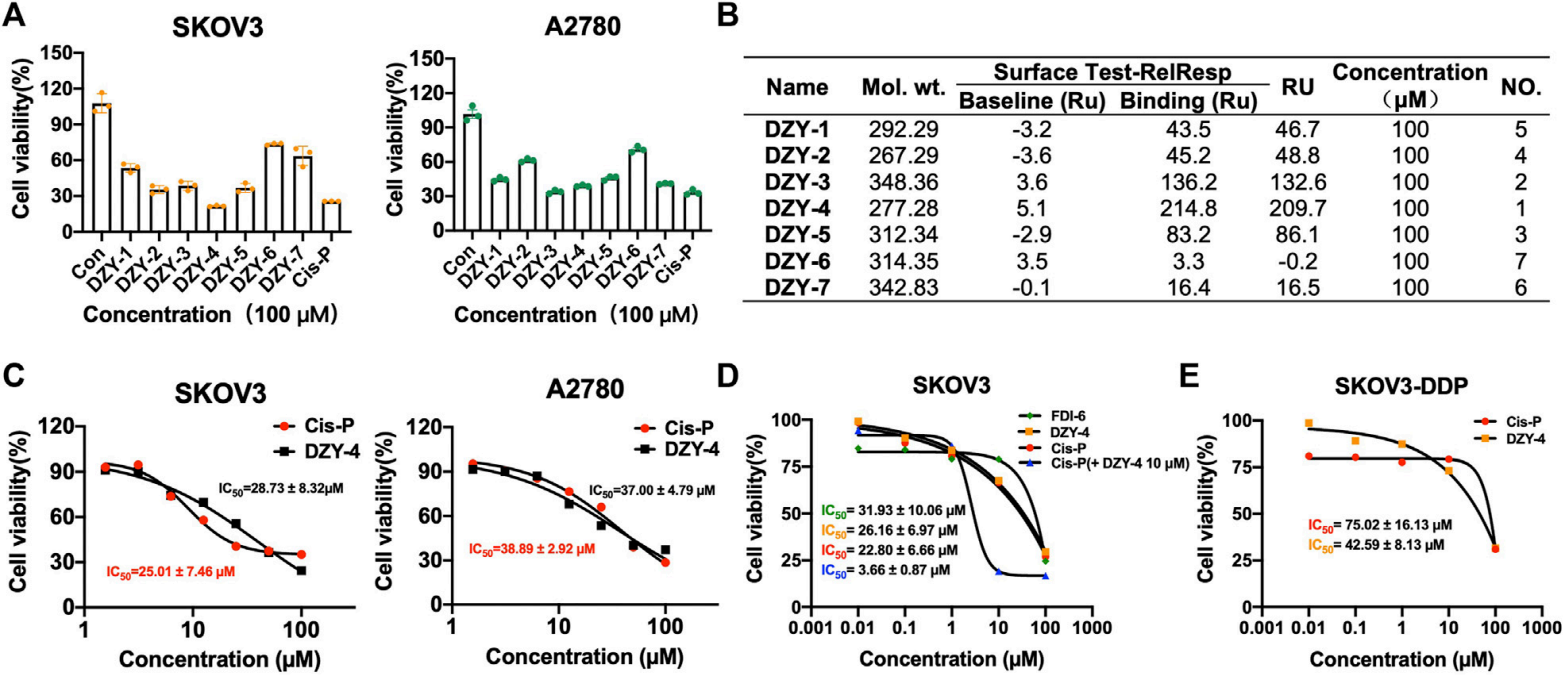
Structure-based virtual screening aimed at discovering and validating FOXM1 inhibitors. **(A)** The effects of FOXM1 inhibitors on the proliferation of SKOV3 and A2780 cells. **(B)** The kinetic interactions of the FOXM1 protein and seven compounds were determined *via* surface SPR analyses. Surface test of small molecules on FOXM1 captured by CM5. **(C)** Effects of **DZY-4** on the proliferation of SKOV3 and A2780 cells. **(D)** IC_50_ values of **FDI-6**, **DZY-4**, **Cis-P**, and **Cis-P** combined with **DZY-4** (10 M) were detected on SKOV3 cells. Data are representative of 3 wells for more than 3 times. **(E)** IC50 values of **Cis-P** and **DZY-4** were detected on drug-resistant SKOV3-DDP cells. Data are representative of 3 wells for more than 3 times.

We purchased the seven candidate compounds obtained *via* virtual screening and evaluated their affinity for FOXM1 at the molecular level *via* surface plasmon resonance (SPR). Anti-tumor activity was also evaluated at the cellular level using the ovarian cancer cell lines, SKOV3 and A2780. The results showed that **DZY-3** and **DZY-4** showed better affinity and antitumor activity at a concentration of 100 µM (Figures 3A,B). **DZY-3** is a camptothecin, which has been used as an antitumor agent, and its new mechanism of action may be found here. However, the off-target effects of **DZY-3** prevent it from acting as a FOXM1-specific inhibitor. The antitumor activity of **DZY-4** and its structural analogs remain unreported. **DZY-4** activity was further evaluated at multiple concentrations, following which the IC_50_ of **DZY-4** were found to be 28.73 µM for SKOV3 and 37.00 µM for A2780 (Figure 3C). The proliferation inhibitory activity of **DZY-4** was better than **FDI-6**. **DZY-4** combined with cisplatin could improve the therapeutic effect of cisplatin (Figure 3D). In SKOV3-DDP cells resistant to cisplatin, **DZY-4** remained active while cisplatin activity decreased significantly (Figure 3E). Based on these results, we propose for the first time that the **DZY-4** class of compounds may exert antitumor effects by inhibiting FOXM1.

### **DZY-4** inhibits the migration and invasion of ovarian cancer cells and induces their apoptosis

**FIGURE 4:**
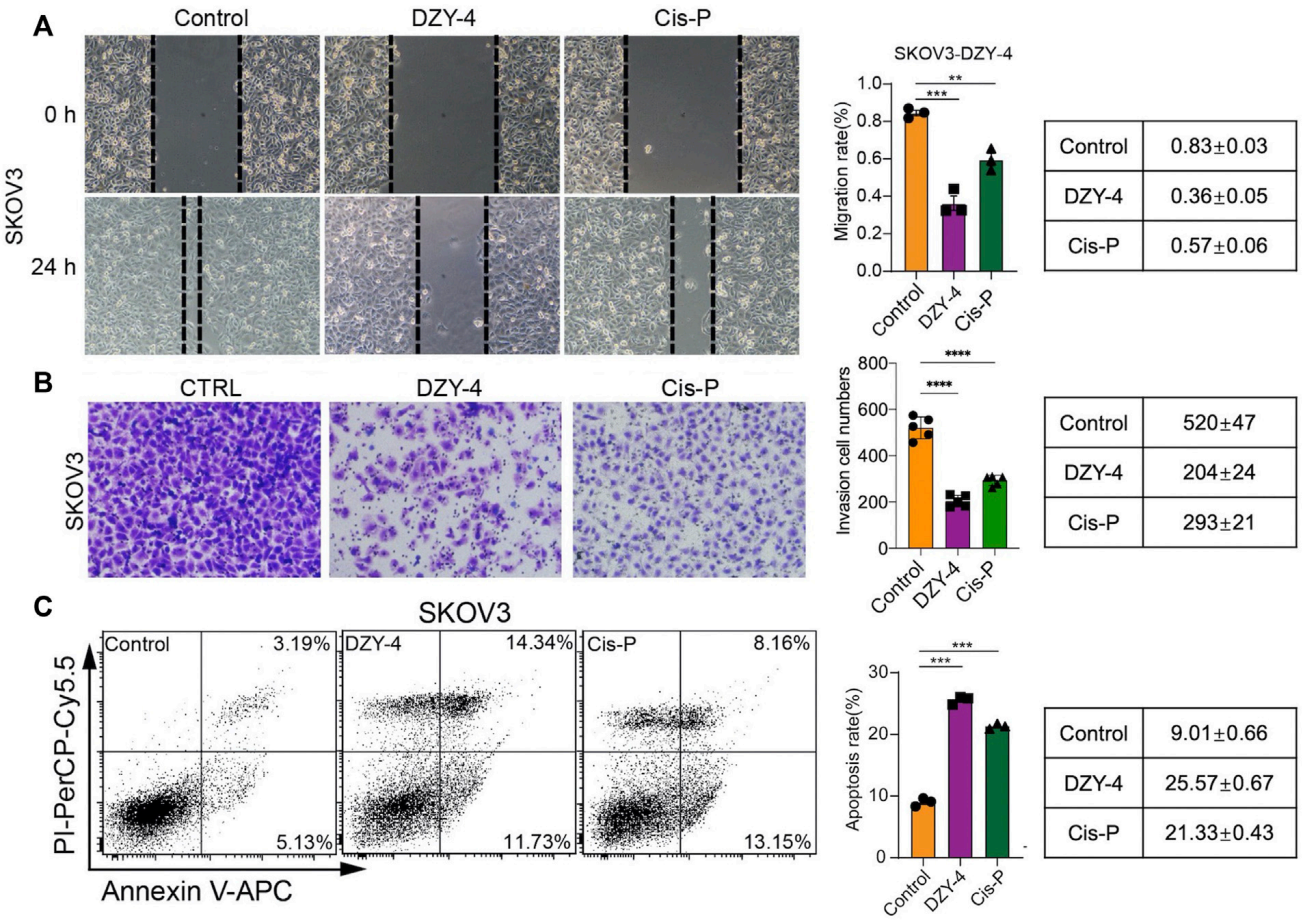
**DZY-4** inhibits the migration and invasion of OC cells and induces their apoptosis. **(A)** Representative images of the migration and quantification of SKOV3 cells. SKOV3 cells were treated with vehicle, **DZY-4** (10 μM), or Cisplatin (10 μM); Scale bar, 50 μm. **(B)** Representative images of invasion and quantification of SKOV3 cells. SKOV3 cells were treated with vehicle, **DZY-4** (10 μM), or Cisplatin (10 μM); Scale bar, 50 μm. **(C)** Representative flow cytometry images of Annexin V-PI-based staining and quantification of apoptosis. SKOV3 cells were treated with vehicle, **DZY-4** (10 μM), or Cisplatin (10 μM). After treatment for 24 h, apoptotic cells were evaluated *via* Annexin V-PI staining (left), histograms showing the statistical results of apoptosis rates (right). The data are expressed as mean ± SEM (*n* = 6 per group). One-way ANOVA was used to compare multiple groups; * = *p* < 0.01, *** = *p* < 0.001, **** = *p* < 0.0001.

We evaluated the anti-ovarian cancer effect of **DZY-4** at the cellular level, using a wound healing assay, an invasion assay and an apoptosis assay, which assessed the effect of **DZY-4** on migration and invasion of SKOV3 cells. Flow cytometry analysis with Annexin V-PI staining was used to evaluate the percentage of apoptotic cells among the SKOV3 cells treated with **DZY-4**. **DZY-4** decreased the migration (Figure 4A) and invasion (Figure 4B) of SKOV3 cells and significantly increased the percentage of apoptotic cells (Figure 4C). The above *in vitro* results indicated that **DZY-4** exerted an anti-ovarian cancer effect which was somewhat superior to that of cisplatin.

### **DZY-4** suppressed ovarian cancer tumor growth *in vivo*

**FIGURE 5:**
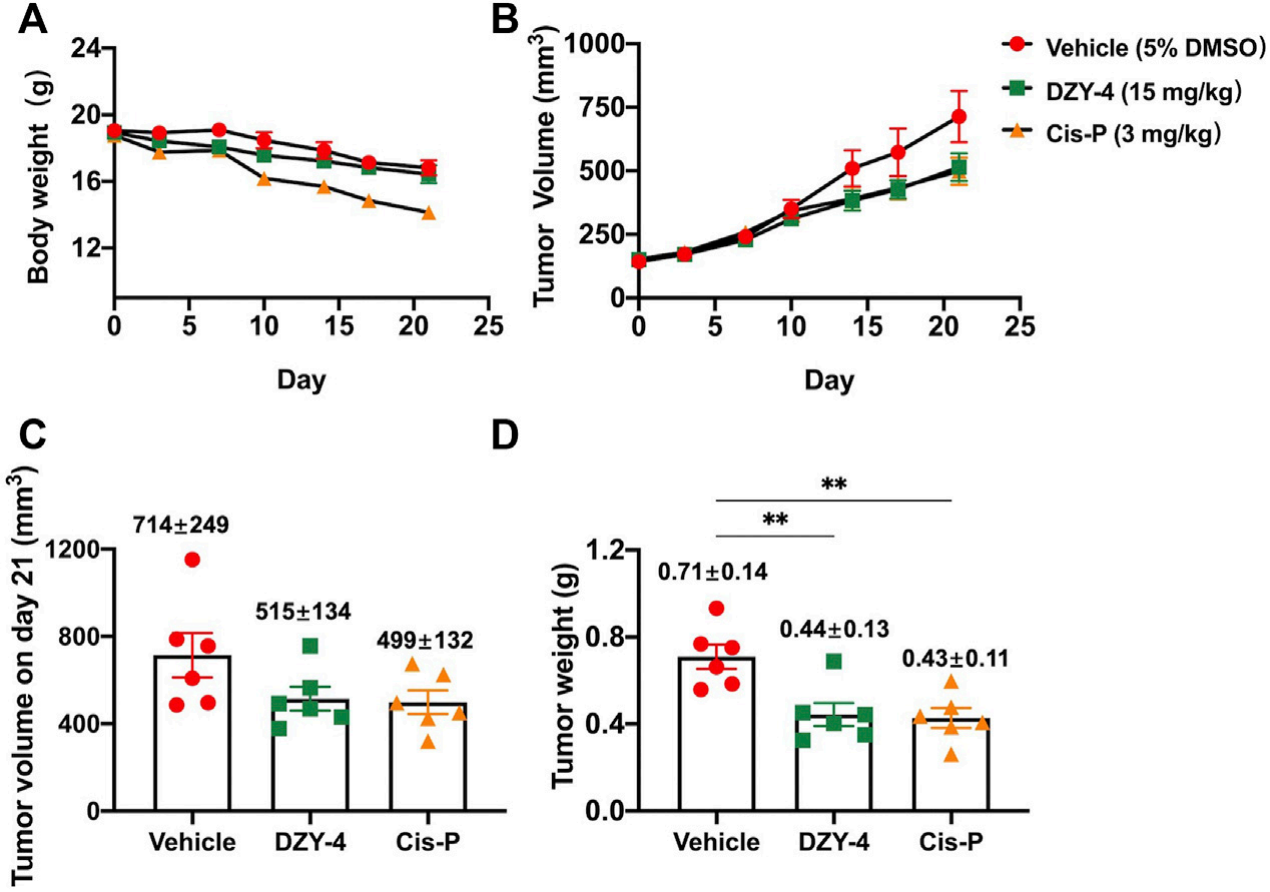
**DZY-4** suppressed OC tumor growth *in vivo*. Nude mice were injected subcutaneously with SKOV3 cells. When the tumor volume reached approximately 100 mm^3^, the mice were treated with vehicle, **DZY-4** (15 mg/kg/day), or Cisplatin (3 mg/kg/week). The data represent quantified body weight **(A)**, tumor volume **(B)**, tumor volume on day 21 **(C)**, and tumor weight **(D)**. The data are expressed as mean ± SEM (*n* = 6 per group). One-way ANOVA was used to compare multiple groups; ** = *p* < 0.01.

Next, we examined the antitumor activity of **DZY-4** in nude mice bearing SKOV3 tumor xenografts. Mice were administered with vehicle, **DZY-4**, or cisplatin *via* intraperitoneal injection (i.p.) for 21 days after the tumor volume reached approximately 100 mm^3^. The weight loss induced by **DZY-4** in tumor-bearing nude mice was consistent with that in the vehicle group, which may be attributed to tumor burden (Figure 5A) Mice in the cisplatin group lost the most weight, indicating that **DZY-4** may be less toxic than cisplatin. Tumor volume was measured during the treatment (Figure 5B). In the *in vivo* efficacy study, **DZY-4** was administered at a significantly higher dose than cisplatin, and the tumor volume on day 21 (Figure 5C) and tumor weight (Figure 5D) were similar in both groups, and both were significantly lower than the blank control group, which had a definite therapeutic effect in ovarian cancer. The final body weight of mice at this dose of **DZY-4** was higher than that of cisplatin, and it may be a lower toxicity than cisplatin.

### **DZY-4** activity was specific to FOXM1 and its homologous proteins

Existing literature shows that the DNA-binding domain of FOXM exhibits 45% identity with those of four of the closest related Forkhead families, namely FOXA, FOXC, FOXO, and FOXP (Laoukili et al., 2007; Li et al., 2017). We selected five proteins with high homology, and based on a literature review, used the conserved residues on H3 helix interacting with DNA as an active site. We also used the “From Receptor Cavities” module based on protein cavities to search for potential binding sites. Then we used LIBDOCK, CDOCKER, and other docking methods to dock **DZY-4** to each of these regions, to generate corresponding complexes (Clark et al., 1993; Stroud et al., 2006; Li et al., 2017; Chen et al., 2019; Li et al., 2021). A comparative analysis showed that CDOCKER was the best method for docking **DZY-4** and the five homologous proteins. The default parameters of CDOCKER Maximum Bad Orientations and Orientation vdW Energy Threshold were 800 and 300, respectively (Table 2). However, due to poor binding between **DZY-4** and the best active site of the FOXM1 homologous protein, it was unable to enter the binding cavity within a general range. Therefore, in order to produce more docking poses, the threshold of the two parameters was adjusted to 999 for CDOCKER docking. Although the threshold for binding between **DZY-4** and homologous proteins was adjusted, **DZY-4** still showed the lowest interaction energy with FOXM1, indicating that **DZY-4** and FOXM1 bind more closely and have a better affinity. In addition, the internal energy of the ligand molecule **DZY-4** and FOXM1 was the lowest (10.6982 kcal/mol). The homologous proteins and **DZY-4** had twice the internal energy. This indicates that **DZY-4** binds more stably to FOXM1. In conclusion, **DZY-4** has the best affinity to FOXM1 and binds it stably, whereas it displayed only a slight binding force with its homologous protein, indicating that **DZY-4** displays specificity for FOXM1.

**TABLE 2:** Energy values of DZY-4 combined with Forkhead box (FOX) proteins.

Name	PDB ID	Energy^a^ (kcal/mol)	Interaction-energy^b^ (kcal/mol)	Maximum bad orientations^c^/Orientation vdW energy threshold^d^
FOXM1	3G73	−18.1145	−28.8127	300/800
FOXA3	1VTN	2.0537	−26.5233	999/999
FOXC2	6AKP	9.5174	−20.2781	999/999
FOXO1	6LBI	2.5111	−22.1046	999/999
FOXA2	5X07	−2.7478	−28.7616	999/999
FOXP2	2A07	−2.0331	−23.7103	999/999

^a^ The sum of the intermolecular interaction energy and the internal energy of the ligand molecule.

^b^ The intermolecular interaction energy.

^c^ The threshold of Van der Waals energy when ligand orientations are adjusted in the active site.

^d^ The maximum number of orientations allowed to be investigated by the program when ligand orientations are adjusted.

## Conclusion

Ovarian cancer is the third most common gynecologic malignancy in terms of incidence; however, it has the highest mortality rate among gynecological malignancies. Most ovarian cancer patients receive first-line chemotherapy consisting of platinum and paclitaxel regardless of whether they qualify for surgery. The occurrence of drug resistance is the main reason for chemotherapy failures. Studies have shown that FOXM1 expression is increased in cisplatin-resistant cancer tissues, and that FOXM1 promotes the malignant behavior of ovarian cancer and induces cisplatin chemoresistance. FOXM1 expression is increased in cisplatin-resistant ovarian cancer cells, and inhibition of FOXM1 expression sensitizes cisplatin-resistant ovarian cancer cells. In summary, ovarian cancer requires urgent improvements in the area of patient prognoses. FOXM1 shows potential as a new therapeutic target in ovarian cancer, where inhibition of FOXM1 is expected to reverse or delay platinum resistance, thereby prolonging PFS and overall survival (OS) in patients. Therefore, the development of high-quality FOXM1 inhibitors as drug leads should be regarded as an important health need.

The specific small molecule FOXM1 inhibitors that have been studied most extensively at the clinical level are **FDI-6** and **RCM-1**, both of which were identified *via* high-throughput screening. **FDI-6** is highly cytotoxic which limits its use as a drug lead. Since the mode *via* which a ligand binds to FOXM1 remains unclear, structural modification of a drug lead cannot be guided. Our team determined the SPR affinity and antitumor activity of **FDI-6** derivatives and combined these with the energy value of docking conformation, to obtain the conformation of a small molecule binding to FOXM1 with high confidence, by analyzing the correlation between these three factors. Based on the above conformation, we conducted virtual screening of over 260,000 compounds in the NCI library, while considering the properties under Lipinski and Veber rules as well as toxicity prediction results of DS software, in an attempt to obtain FOXM1 inhibitors with new structural backbones and better drug-forming properties. The results showed that six of the seven selected compounds showed an affinity to the FOXM1 protein, corroborating the accuracy of the conformation.

Among the seven compounds selected, **DZY-3** and **DZY-4** showed the best affinity for FOXM1 and the best inhibitory activity against two ovarian cancer cell lines, SKOV3 and A2780. **DZY-3**, (camptothecin), which inhibits DNA replication in proliferating tumor cells, has been used as an antitumor drug for many years, and this study was able to propose a new mechanism for its antitumor action, by demonstrating that it inhibits FOXM1. **DZY-4** showed the highest affinity to FOXM1 and its IC_50_ values for both ovarian cancer cells were comparable to those of cisplatin. **DZY-4** reduced tumor cell migration and invasion and increased the percentage of apoptotic cells. Although a dose of **DZY-4** greater than that of cisplatin was used to treat the nude mouse subcutaneous transplantation tumor model, weight loss was lower than that of cisplatin and the tumor volume and tumor weight were comparable to those of cisplatin. Virtual molecular docking studies showed that **DZY-4** was specific for FOXM1, when compared to its homologous proteins. The antitumor activity of **DZY-4** and its structural analogs has not been reported in the literature to date; to the best of our knowledge, the current study is the first to report the FOXM1 inhibition-based antitumor effect of such structures of **DZY-4**.

In summary, **DZY-4** shows potential as a therapeutic agent for ovarian cancer and may be used as a drug lead for further studies and as a biochemical research tool to promote studies investigating FOXM1 as a drug target. In the future, we aim to conduct drug discovery tests based on **DZY-4** and search for derivative directions to guide conformational studies that may enhance the affinity of the docking conformation of **DZY-4** for FOXM1. This study is expected to promote the search for ovarian cancer therapeutic drugs guided by new mechanisms.

## Materials and methods

### Molecular docking

Ligands of the NCI diversity Set2 database were docked with FOXM1 using AutoDock Vina package. AutoDock Tools 1.5.6 was used to compile the grid and docking parameter files. The active site was defined as 12.963 Å × 31.496 Å × 2.757 Å. The docking grid size was defined as 26, 16, and 20 Å on the X, Y, and Z coordinates respectively. A hydrogen atom with a pH of 7.4 was added to the ligand through the babel module. Next, the conformation was searched using OBConformer Search and Monte Carlo simulations, following which energy was optimized using the steepest descent method with a maximum of 100 steps.

The X-ray structure of FOXM1 and its homologous proteins (PDB ID: 3G73, 1VTN, 6AKP, 6LBI, 5X07, 2A07) were downloaded from the Protein Data Bank (https://www.rcsb.org). The proteins were prepared according to standard operations. The co-crystallized water molecules were deleted, and the “Clean Protein” and “Prepare Protein” tools of Discovery Studio were used to resolve potential problems in protein structures, such as the model missing loop regions, delete alternative conformations, add hydrogen atoms, and generate the protonation state at pH 7.0. The active site was identified according to previous reports or studies, and the docking radius of the sphere was set to 9 Å. The compounds were “Full Minimization.” Discovery Studio 4.0 was used to carry out the CDOCKER method to perform docking-based virtual screening.

### Cells and cell culture

SKOV3 and A2780 cells were purchased from Procell Life Science and Technology Co., Ltd. The cells had recently been authenticated by short tandem repeat (STR) profiling and characterized by mycoplasma and cell viability detection. A2780 cells were cultured and maintained in MEM (Gibco, Catlog No. 11090081) supplemented with 10% fetal bovine serum (FBS; Gibco, Catalog No. 10091148) under 5% carbon dioxide. SKOV3 cells were cultured and maintained in DMEM (Gibco, Catalog No. C11885500BT) supplemented with 10% FBS.

### Xenografts

All animal studies were approved by the Animal Experimentation Ethics Committee of the Chinese Academy of Medical Sciences, and all procedures were conducted in accordance with the guidelines of the Institutional Animal Care and Use Committees of the Chinese Academy of Medical Sciences. Five-week-old female BALB/c nude (Weitong Lihua) mice were used to determine the drug effects of DZY-4 *in vivo*. For subcutaneous xenograft models, 1 × 10^6^ SKOV3 cells were collected, resuspended in PBS, and injected into the left flanks of nude mice. When the tumors reached a volume of 100 mm^3^, the mice were randomly divided into three groups (*n* = 6). DZY-4 (15 mg/kg, daily) or cisplatin (3 mg/kg, once per week) in DMSO (5%) was administered *via* i.p. injection for 21 days. A digital caliper was used to gauge tumor size, and tumor volume was calculated using the formula: tumor volume (mm^3^) = width^2^ × (length/2).

### Cell Counting Kit-8 assay

For the Cell Counting Kit-8 (CCK-8) assay, A2780 and SKOV3 cells were seeded in 96-well plates at a density of 3,000 cells/well. For verification of FOXM1 inhibitors regarding the proliferation of A2780 and SKOV3 cells, Cisplatin (100 μM) and FOXM1 inhibitor (100 μM) treated cells were cultured for 24 h. For the effects of DZY-4 on the proliferation of A2780 and SKOV3 cells, DZY-4 (0.78, 1.56, 3.125, 6.25, 12.5, 25, 50, 100 μM) or Cisplatin (0.78, 1.56, 3.125, 6.25, 12.5, 25, 50, 100 μM) treated cells were cultured for 24 h. Subsequently, 100 μl of complete medium supplemented with 10 μl of CCK-8 solution (TOPSCIENCE, C0005) was added to each well, and the plates were incubated for 2 h. Finally, absorbance was measured at 450 nm.

### Surface plasmon resonance analysis

Surface plasmon resonance (SPR) assays were performed using a Biacore T200 (GE Healthcare) at 25°C. Recombinant FOXM1 (amino acids 222–360) protein [Purity (SDS page) > 90%] was purchased from Genescript USA Inc. (NJ, China) and covalently immobilized on a CM5 sensor chip (GE Healthcare) *via* amine coupling. Running buffer was 1.0 × PBS + P (GE Healthcare). When the affinity screen of fragments was run, 5% DMSO was added to the buffer. Compounds were injected into the surface of the protein-coupled chip channels at the flow rate of 30 µl/min. The affinities of the fragments were tested once in this study.

### Wound healing assays

SKOV3 cells were harvested and seeded into 12-well plates at 2 × 10^5^ cells per well. When cells reached 90% confluence, they were scratched with a 200 μl pipette tip to create a wound and washed with PBS to remove cells that had detached. The cells were then treated with DZY-4 (10 μM) or Cisplatin (10 μM), respectively, for 24 h. Cells were imaged with an inverted microscope (Olympus, Tokyo, Japan). Scratch healing efficiency was analyzed by calculating trauma distance.

### Invasion assays

A transwell chamber (Millipore, SCWP04700) with an 8 μm pore size filter membrane was used for the invasion experiments. The Matrigel (40 μl per well, BD Matrigel Matrix, 354230) was coated with 10 μg/ml FN1 (fibronectin 1; MERCK, F0635), polycarbonate filter. Subsequently, the chambers were inserted into 24-well culture plates. Single-cell suspensions were inoculated into the upper chamber (5 × 10^4^ cells per well in 0.4% FBS in DMEM). Twelve hours later, uninvaded cells in the upper part of the filter were removed with a cotton swab. Invading cells were fixed in 4% paraformaldehyde in PBS, stained with 0.1% crystal violet, and counted in six random fields *via* bright-field microscopy.

### Apoptosis

Cell apoptosis was analyzed using a FITC Annexin V Apoptosis Detection Kit I (catalog 55647, BD Pharmingen). SKOV3 cells were treated with DZY-4, Cisplatin, or DMSO. A total of 10^5^ cells were collected after DZY-4 exposure and resuspended in a 1 × binding buffer. Five microliters of FITC Annexin V and 5 μl PI were then added to the cell suspension and incubated for 15 min at room temperature in the dark. The results were determined *via* flow cytometry within 1 h.

### Statistical analysis

Data are expressed as the mean ± SEM. For experiments involving only 2 comparisons, a 2-tailed Student’s t-test was used. For experiments involving multiple comparisons, the significance of differences between treatment means was determined using ANOVA followed by a Bonferroni multiple-comparison test. All analyses were performed using GraphPad Prism 9 (GraphPad Software). *p* values less than 0.05 were considered statistically significant. For all figures, * = *p* < 0.05, ** = *p* < 0.01, *** = *p* < 0.001.

## Data Availability

The original contributions presented in the study are included in the article/Supplementary Material, further inquiries can be directed to the corresponding authors.
